# The Asparaginase Database: a comprehensive resource and classification of l-asparaginases

**DOI:** 10.1093/database/baag045

**Published:** 2026-07-31

**Authors:** Max Štětina, Aleš Křenek, Guglielmo Tedeschi, Filip Krása, Vojtěch Spiwok, Eva Benešová

**Affiliations:** Department of Biochemistry and Microbiology, University of Chemistry and Technology, Prague, Technická 3, Prague 6, 166 28, Czech Republic; Institute of Computer Science, Masaryk University, Šumavská 416/15, Brno, 602 00, Czech Republic; Department of Biochemistry and Microbiology, University of Chemistry and Technology, Prague, Technická 3, Prague 6, 166 28, Czech Republic; Institute of Computer Science, Masaryk University, Šumavská 416/15, Brno, 602 00, Czech Republic; Department of Biochemistry and Microbiology, University of Chemistry and Technology, Prague, Technická 3, Prague 6, 166 28, Czech Republic; Department of Biochemistry and Microbiology, University of Chemistry and Technology, Prague, Technická 3, Prague 6, 166 28, Czech Republic

## Abstract

l-Asparaginases have been essential anticancer biopharmaceuticals for nearly half a century, particularly in the treatment of acute lymphoblastic leukaemia. Despite their success, current preparations face challenges, such as strong immunogenicity, reduced stability, and secondary l-glutaminase activity. Systematic discovery and engineering of l-asparaginases with desirable properties are hindered by the limitations of existing l-asparaginase classifications being unable to keep pace with rapid sequence discovery or not reflecting the growing diversity of l-asparaginase characteristics. We present The Asparaginase Database, a resource for curated l-asparaginase sequences, conserved motifs, structures, and experimental data organised by a sequence similarity driven classification into 40 phylogenetic families and 14 clans within previously established l-asparaginase classes. This framework provides a new model for the classification of l-asparaginases based on large-scale molecular phylogeny and could enable researchers to navigate l-asparaginase diversity and identify promising candidates for therapeutic development. **Database URL:**  https://asparaginasedb.com/

## Introduction

l-Asparaginases are a group of enzymes that catalyse the hydrolysis of l-asparagine to l-aspartate and ammonium. This reaction is important in nitrogen metabolism and maintaining cellular and physiological homeostasis [1]. l-Asparaginases are found in all domains of life, and sequences are commonly present in several isoforms. Consequently, they emerge as a highly diverse group, characterised by diverse quaternary structures and distinct amino acid sequences, while sometimes accepting various substrates beyond l-asparagine [1,2].

l-Asparaginases (EC 3.5.1.1) have become an integral part of chemotherapeutic regimens for paediatric patients with haematological malignancies, particularly acute lymphoblastic leukaemia. These malignant cells lack the ability to adequately synthesise l-asparagine *de novo* and depend on extracellular sources [3]. Survival rates of children with acute lymphoblastic leukaemia rose from 10% to 90% following decades of improvements in multi-agent therapeutic regimens [4], which include l-asparaginase. Furthermore, l-asparaginase therapies could have potential in the treatment of some solid tumours [5].

However, many patients suffer from severe side effects, such as hepatotoxicity, pancreatitis, thrombosis, and allergic reactions [6]. Therefore, the development of l-asparaginases with reduced immunogenicity, improved stability, and high affinity and specificity for l-asparagine remains crucial for minimising toxicity in treatments. As clinically approved l-asparaginases are of bacterial origin (*Escherichia coli* and *Dickeya dadantii*), exploring enzymes from alternative sources may offer distinct therapeutic advantages, both to improve current treatments and to expand into other applications where tolerability issues have limited their clinical exploration [7].

Another important application of l-asparaginases is in the food industry, where they are used to lower acrylamide levels in carbohydrate-rich heat-processed foods such as fried potato products and bakery products [8]. The neurotoxic effects of acrylamide are well-studied [9] and it is classified as a probable carcinogen [10]. The hydrolysis of l-asparagine prevents it from reacting with reducing sugars at high temperatures and creating acrylamide in the process known as the Maillard reaction. Ongoing research is focused on developing improved or characterising novel l-asparaginases with properties tailored for safer food processing and biosensors [11]. This research gains additional relevance as the European Union has been working towards legally binding maximum levels for acrylamide (European Commission, ‘Acrylamide’, https://food.ec.europa.eu/food-safety/chemical-safety/contaminants/catalogue/acrylamide_en, accessed 20 June 2026).

Classification is central to understanding, discovering, and developing l-asparaginases. Historically, these enzymes have been divided into three families: bacterial-type, plant-type, and *Rhizobium etli*-type [12], named after representative members of each group. This nomenclature can be a source of confusion, as the sequences in each family commonly originate from organisms across multiple domains. The bacterial-type family was further subdivided into type I (cytoplasmic) and type II (periplasmic) l-asparaginases. This subdivision stems from the early characterisation of l-asparaginase isoforms from *E. coli*, which served as the model system [13].

To update this, da Silva *et al*. [2] proposed a new classification based on peptide domains, phylogenetic data, structural analysis, and functional characteristics. Their system defines three classes that parallel the historical families but avoids taxonomic labels: Class 1 (formerly bacterial-type), Class 2 (plant-type), and Class 3 (*R. etli*-type). Classes 1 and 2 are subdivided into four and three groups, respectively, organised according to distinct features. However, the phylogenetic analysis was performed on 337 manually selected sequences, while sequence homology searches identify over 100 000 unique putative l-asparaginases.

Following this, Loch and Jaskolski [1] proposed an alternative classification that expands on the historical model. They endorsed the detachment from biological taxonomy but argued against renaming historically established enzymes. While this system addresses the taxonomic nomenclature issue, it relies on a limited set of model enzymes that may not represent the full diversity of their respective families. Table 1 sets the two systems alongside each other for a selection of representative sequences.

**Table 1:** Representative l-asparaginase and related sequences mapped to both the da Silva *et al.* [2] and Loch and Jaskolski [1] classification systems

				da Silva *et al*. [2]		Loch and Jaskolski [1]	
UniProt ID	Short name	UniProt protein name	Organism	Class	Group	Type^a^	Sub-type
P00805	EcAII	l-asparaginase 2	*Escherichia coli*	Class 1	Group 1	bacterial	type 2
P38986	ScAI^b^	l-asparaginase 1	*Saccharomyces cerevisiae*		Group 2		
Q9V0T9	–	Glutamyl-tRNA^Gln^ amidotransferase subunit D	*Pyrococcus abyssi*		Group 3		type 1
P0A962	EcAI	l-asparaginase 1	*Escherichia coli*		Group 4		
P37595	EcAIII	Isoaspartyl peptidase	*Escherichia coli*	Class 2	Group 1	plant	type 3
Q9H6P5	–	Threonine aspartase 1	*Homo sapiens*		Group 2		
P20933	–	*N*(4)-(β-*N*-acetylglucosaminyl)-l-asparaginase	*Homo sapiens*		Group 3		
Q2KB35	ReAIV	l-asparaginase II^c^ protein	*Rhizobium etli*	Class 3	–	*R. etli*	type 4
Q2K0Z2	ReAV	l-asparaginase II^c^ protein	*Rhizobium etli*				type 5

Sequences not explicitly classified by Loch and Jaskolski [1] were assigned based on sequence similarity to those that were classified.

^a^Although Loch and Jaskolski [1] adopted the class nomenclature, this table contains the historical types for context.

^b^The *S. cerevisiae* ScAI (P38986) was sometimes considered a type 1 (or type I) l-asparaginase; however, the sequence is more similar to type 2 l-asparaginases.

^c^The protein name l-asparaginase II is unrelated to the type 2 (or type II) classification of l-asparaginases.

These limitations motivated the development of a classification system based on comprehensive sequence analyses: scalable, unbiased, and encompassing all known l-asparaginase and l-asparaginase-like sequences identified by homology alongside characterised enzymes. To this end, we developed an l-asparaginase database that integrates sequences, structures, and experimental data within the comprehensive classification.

## Materials and methods

The UniRef100 database (Release 2023_03) [14] was selected as the primary sequence source for the bioinformatic analysis underlying the classification due to its comprehensive coverage and non-redundancy. The well-annotated sequences from Swiss-Prot (Release 2023_02) [15] served as reference points for the classification.

The Protein Data Bank (PDB) [16] was accessed through the European Bioinformatics Institute website on 17 October 2023 and subsequently monitored for l-asparaginases with experimentally determined structures. Structures in the AlphaFold Protein Structure Database [17,18] were analysed when experimentally solved structures were not available. Structures were further identified and compared with Foldseek release 10 [19].

The literature was systematically searched for experimentally characterised enzymes whose biochemical data could be linked to a specific sequence.

To identify l-asparaginase sequences for analysis, the Swiss-Prot and UniRef100 databases were searched for l-asparaginases with BLASTP version 2.10.0+ [20]. The default settings were used, except the E-value, which was set to 10^–3^ for the Swiss-Prot search and to 10^–4^ for the UniRef100 search and used as the defining cut-off. Query sequences were selected from each group in the da Silva *et al*. [2] classification, providing the basis for broader similarity-based discovery. The selected query sequences, categorised according to previous classifications, are listed in Table 1 (except ReAIV, which was omitted as a query sequence due to near-complete redundancy with ReAV in Class 3). BLAST results from queries within each class were combined separately for each database, and duplicate sequences resulting from multiple queries per class were removed. No sequence overlap was observed between classes.

Sequences retrieved from UniRef100 were clustered using CD-HIT version 4.8.1 [21] at 80% sequence identity; other parameters remained at default settings. Next, Swiss-Prot and clustered UniRef100 representatives were pooled. Although UniRef100 incorporates Swiss-Prot sequences, clustering at 80% identity often selected non-Swiss-Prot sequences as cluster representatives, necessitating manual readdition of Swiss-Prot sequences to preserve well-annotated reference points in the alignment. Alignments were generated with Fast and Accurate Multiple Sequence Alignment (FAMSA) version 2.2.2 [22] with default settings. Approximate maximum-likelihood phylogenetic trees were inferred using FastTree version 2.1.11 [23] with default settings for protein sequences.

After individual phylogenetic trees were constructed for each class, Swiss-Prot sequences, solved structures, and experimentally studied proteins were mapped onto the trees. Branch lengths and local support values were analysed in iTOL v6 [24] to define initial family boundaries. Each putative family was taxonomically profiled using UniProt ID mapping. Sequences within each initial family were clustered at 70% sequence identity using CD-HIT with otherwise default settings, and 100 sequences were randomly sampled. These subsets were aligned using Clustal Omega version 1.2.4 [25] with default parameters, and alignments were stripped of sequences exhibiting likely sequencing errors and rendered in Jalview version 2.11.5.1 [26]. The conserved sequence motifs were identified and mapped onto representative sequences from each family. The family boundaries were iteratively refined (if necessary), with additional consideration given to oligomeric organisation, structural features, taxonomic origin, and catalytic function when such data were available. Finally, phylogenetically related families were grouped into clans.

The Asparaginase Database (https://asparaginasedb.com/) is deployed on a Kubernetes infrastructure running on Linux. Sequence similarity searches (NCBI BLASTP version 2.12.0+) are executed with a Python FastAPI backend, enabling users to search separately in the full database, experimentally studied proteins, and Swiss-Prot entries. Website development was assisted by Claude from Anthropic. The infrastructure includes maintenance and update capabilities to ensure data consistency across the hierarchical classification system. We plan to semi-automatically update the database twice a year. Its maintenance is supported by ELIXIR Czech Republic.

## Results and discussion

The database covers a manually curated set of 101 experimentally studied proteins from the literature (including 32 with solved structures), 127 manually curated sequences from Swiss-Prot, and 126 085 classified sequences from UniRef100. The database contains not only l-asparaginases, but also evolutionarily related enzymes such as mixed l-asparaginases/l-glutaminases, β-aspartyl-peptidases, Glu-tRNA^Gln^ amidotransferases, aspartyl-glucosaminidases, and others. Users can browse entries with kinetic and structural data, search and filter the database by various parameters, classify sequences or identify the closest experimentally characterised proteins using BLAST, download organised data and sequences, and access linked source publications.

**Figure 1:**
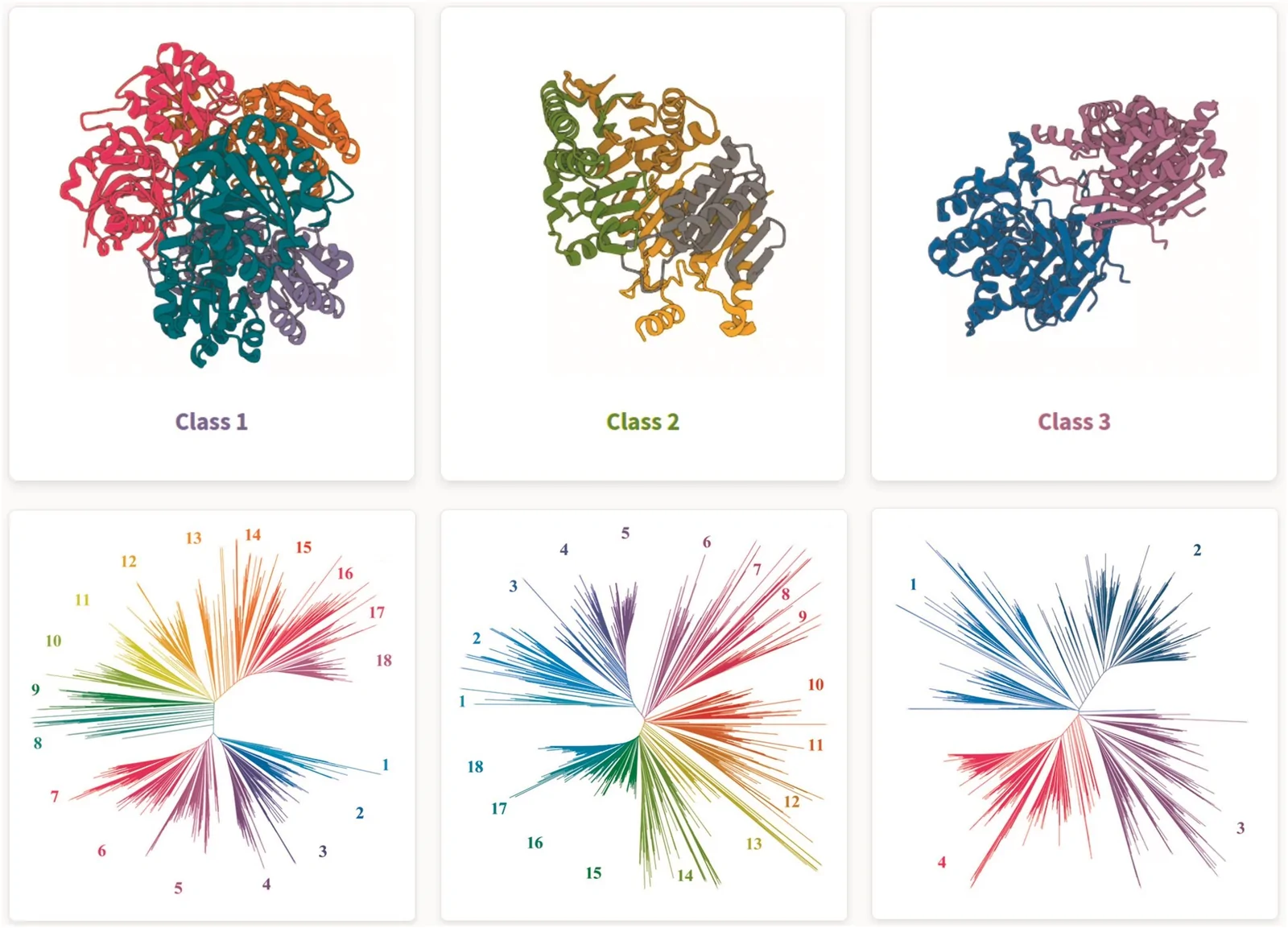
Illustrative structures from the front page of the database pointing to classes 1, 2, and 3 with the corresponding phylogenetic trees; numbers indicate the phylogenetic families within each class.

The enzymes are classified into three classes previously established in the literature, now further divided into 14 clans consisting of 40 phylogenetic families (Fig. 1). This hierarchical structure is analogous to the CAZy database [27] for carbohydrate-active enzymes, which classifies enzymes on the basis of sequence and structure rather than substrate specificity. Detailed class and family pages contain additional information (e.g. taxonomic origin, substrate specificity), phylogenetic trees, representative sequence alignments, and conserved motifs.

As the classification is based on molecular phylogeny, it is more objective and scalable. While the previous phylogenetic classification by da Silva *et al*. [2] examined 337 manually selected sequences, our classification is by far the most comprehensive to date. This integration of classification and online database also allows users to easily determine the class, clan, and family assignment of any analysed sequence, as well as novel sequences with similarity searches.

Within this classification, many families consist almost exclusively of enzymes originating from a single taxonomic group, while others are taxonomically diverse, likely due to horizontal gene transfer events. Other trends, such as thermal stability, high affinity for l-asparagine, or presence of additional domains, can be observed but are not always correlated with individual families, reflecting the complex evolutionary history of these enzymes.

The classification avoids historical nomenclature issues, while the database provides historically assigned protein names for context. Most phylogenetic families are precisely defined by evolutionary distance and local support values, supplemented by experimental, structural, and taxonomic context, although a few remain weakly supported, and some sequence placements within these should be considered tentative. Unlike the manually curated entries, sequences from UniRef100 were not filtered before inclusion and may contain sequencing errors or represent non-functional proteins; we recommend comparing sequences to the alignments and conserved motifs of the relevant families before further use. Many families also lack experimentally studied representatives, presenting opportunities for characterising enzymes that may exhibit novel properties or contain previously unreported l-asparaginase conserved motifs. Not all characterised l-asparaginases in the literature are included, often due to publications lacking sufficient data for inclusion or classification of the sequence.

## Conclusion

The Asparaginase Database is the first dedicated resource for l-asparaginases and related proteins organised by large-scale phylogenetic classification, enabling researchers to search, classify, and explore the sequence, structural, and kinetic landscape of these enzymes.

## Data Availability

The data underlying this article are available in The Asparaginase Database at https://asparaginasedb.com/, and are released under a CC BY 4.0 license.
